# Notoginsenoside R7 suppresses cervical cancer via PI3K/PTEN/Akt/mTOR signaling

**DOI:** 10.18632/oncotarget.22721

**Published:** 2017-11-27

**Authors:** Li Li, Jin-Xia Sun, Xiao-Qi Wang, Xiao-Kai Liu, Xian-Xiong Chen, Bo Zhang, Zhen-Dan He, Dong-Zhou Liu, Li-Xin Chen, Li-Wei Wang, Zhong Huang

**Affiliations:** ^1^ Institute of Biological Therapy, Shenzhen University, Shenzhen 518060, China; ^2^ Department of Immunology, Shenzhen University School of Medicine, Shenzhen 518060, China; ^3^ Department of Pharmacy, The Eighth Affiliated Hospital of Zhongshan University, Shenzhen 518000, China; ^4^ Department of Rheumatology & Immunology, Ji’nan University 2nd Clinical Medicine College, Shenzhen People’s Hospital, Shenzhen 518020, China; ^5^ Institute of Traditional Chinese Medicine and Natural Products, College of Pharmacy, Jinan University, Guangzhou 510632, China

**Keywords:** notoginsenoside R7, PI3K/PTEN/Akt/mTOR, apoptosis, proliferation, cervix cancer

## Abstract

Notoginsenoside R7 was isolated from *Panax notoginseng*, a plant used commonly in traditional Chinese medicine. We investigated the anti-cancer effects of R7 in HeLa cells *in vitro* and *in vivo*, and explored the underlying mechanisms of action. R7 dose-dependently inhibited HeLa cell proliferation and induced apoptosis *in vitro*, *In silico* docking-based screening assays showed that R7 can directly bind Akt. Pretreatment with the Akt inhibitor LY294002 synergistically enhanced the R7 anti-proliferation and anti-apoptosis effects in HeLa cells, confirming that R7 acts through the PI3K/Akt pathway. Consistent with the *in vitro* findings, R7 exerted anti-tumor effects in a mouse xenograft model by targeting PI3K (PTEN) and Akt, activating the pro-apoptotic Bcl-2 family and, subsequently, caspase family members. R7 treatment activated PTEN and downregulated mTOR phosphorylation without affecting mTOR expression, though regulatory-associated protein of mTOR (raptor) expression declined. Our study suggests that R7 is a promising chemotherapeutic agent for the treatment of cervical cancer and other PI3K/PTEN/Akt/mTOR signaling-associated tumors.

## INTRODUCTION

Due to their therapeutic efficacies and minimal adverse effects, biologically active natural compounds are gaining popularity as potential first-line treatments [1, 2]. Notoginsenoside R7 (R7, Panaxadiol-3-O-β-D-glucopyranoside) is a triterpenoid saponin isolated from the dried root and rhizome of *Panax notoginseng* (Burk.) F.H.Chen, which is found mainly in south western China. *P. notoginseng* has long been used in traditional Chinese medicine (TCM) as part of the “medicine-food homology” concept, particularly in the treatment of inflammation, swelling, pain, and “blood stasis,” which is considered an underlying pathology for many diseases in TCM [3]. Compounds extracted from *P. notoginseng* exhibit wide-ranging pharmacological activities, including anti-inflammatory, anti-atherosclerotic, anti-hypertensive, hepatoprotective, neuroprotective, and anti-tumour activity [4–6]. The anti-tumor activities of triterpenoid saponins suggest that ginsenosides Rg3, Rg5, and Rh2 could induce cancer cell apoptosis and inhibit invasion, metastasis, and cell cycle progression [7–10]. Mechanistic studies suggest that notoginsenosides downregulate vascular endothelial growth factor (VEGF) expression, inhibit NF-κB activity, and promote autophagy [11]. Our preliminary screening found that R7 has potent anti-tumor properties. To our knowledge, no pharmacological bioactivity has thus far been reported for R7, and the underlying mechanisms of its anti-cancer effects remain unclear.

Cervical cancer is the fourth most common cancer in women globally and the second most common in developing areas [12]. Most patients with cervical cancer receive conventional chemotherapy or radiotherapy, but serious adverse reactions to therapies are not uncommon [13, 14]. Novel effective, less toxic drugs are urgently needed to reduce patient mortality. Inhibition of cell apoptosis is a main cause of tumorigenesis and progression [15, 16], and numerous studies have implicated PI3K/PTEN/Akt/mTOR signaling in regulation of cell apoptosis and proliferation [17, 18]. Compounds that target PI3K/PTEN/Akt/mTOR signaling are of considerable interest as anti-cancer agents, and several have been studied in clinical trials [19, 20].

The PI3K/PTEN/Akt/mTOR pathway is activated through tyrosine-kinase growth factor receptors and small G proteins at the cell membrane [21]. PI3K activation catalyzes phosphatidylinositol-3,4-bisphosphate (PIP2) phosphorylation, which is then converted into phosphatidylinositol-3,4,5-triphosphate (PIP3). PIP3 acts as a second messenger, recruiting and activating Akt through direct binding of Akt PH domains [22]. The best-studied downstream substrate of Akt is mTOR, which can be directly phosphorylated and activated by Akt. Akt/mTOR activation initiates a signaling cascade that regulates cell apoptosis, survival, growth, and proliferation, and can promote tumorigenesis and angiogenesis [17, 18]. The tumor suppressor, PTEN, negatively regulates PI3K/Akt signaling and antagonizes PI3K by dephosphorylating PIP3 to PIP2 [23, 24]. Mice with PTEN deletion or mutation are highly susceptible to tumor development [25], and PTEN downregulation is observed in ovarian, uterine, breast, prostate, and cervical cancers [23, 24, 26]. Additionally, PTEN conditional knockout leads to tumor growth in multiple organs, such as the mammary gland, skin, and prostate [27, 28].Thus, PI3K/PTEN/Akt/mTOR pathway activation may initiate tumorigenesis through the following mechanisms: (i) inhibition of apoptosis; (ii) autophagy; (iii) promotion of metastasis; or (iv) resistance to chemotherapy. Development of new PI3K/PTEN/Akt/mTOR inhibitors is of great clinical interest in cancer treatment.

The present study investigated R7 anti-tumor activity and underlying mechanisms of action. *In silico* docking and virtual screening assays were performed to identify potential R7 targets. The effects of R7 on PI3K/PTEN/Akt/mTOR signaling were examined in HeLa cells, and R7 anti-tumor activity was confirmed in a mouse xenograft model. Our findings demonstrate for the first time that R7 induces apoptosis through PI3K/PTEN/Akt/mTOR inhibition, and may be a promising candidate in the clinical treatment of cervix cancer.

## RESULTS

### R7 suppresses HeLa cell proliferation

**Figure 1:**
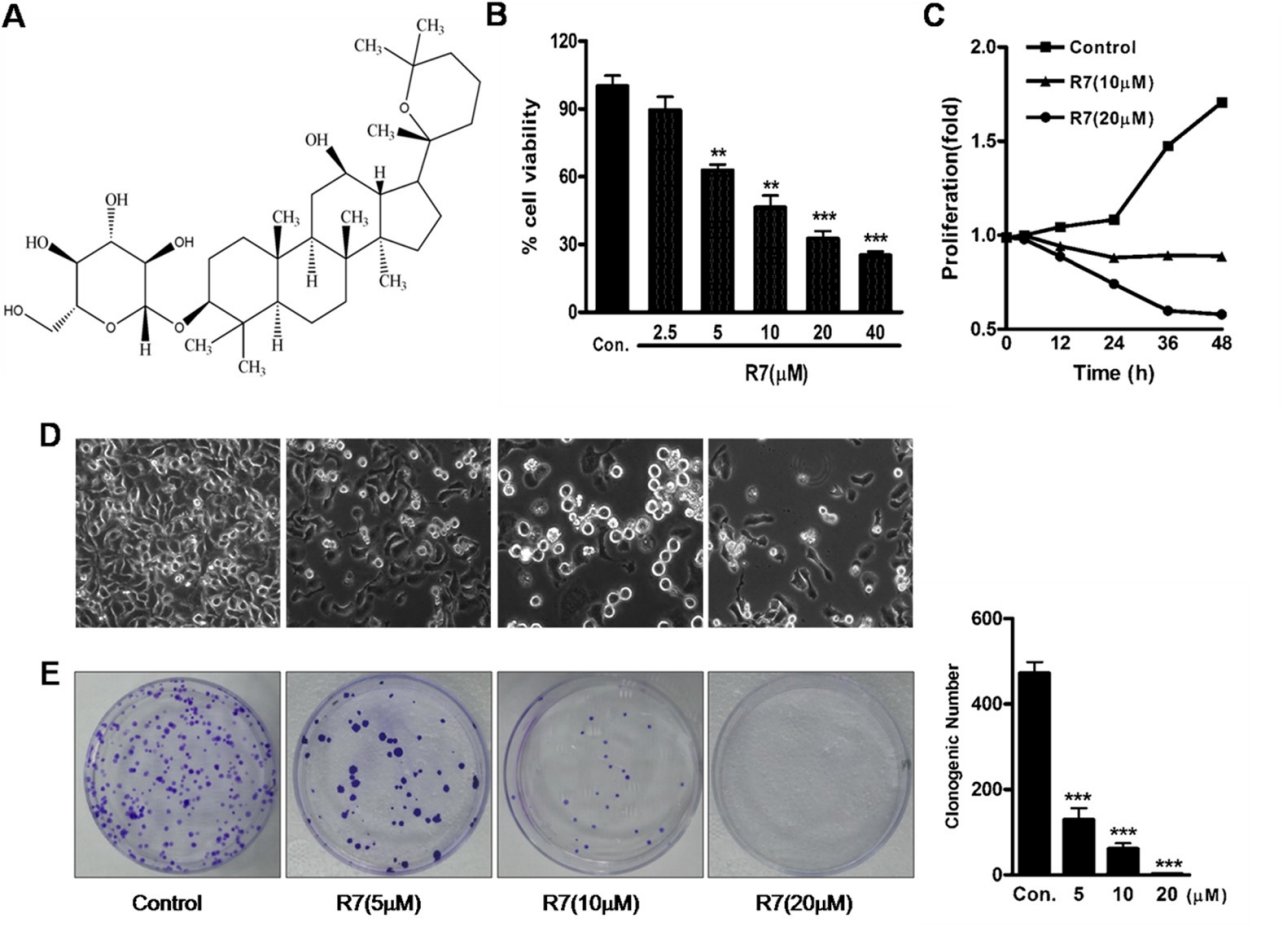
R7 inhibits HeLa cell proliferation The chemical structure of R7. **(A)** R7 (2.5, 5, 10, 20, or 40 μM) 24 h dose response in HeLa cells. **(B)** Cell proliferation was measured by MTT assay. R7 inhibits HeLa cell proliferation in a time-dependent manner. **(C)** Percentage of viable cells at 0, 12, 24, 36, and 48 h relative to controls. Effect of R7 on morphological changes as shown by phase-contrast microscopy. **(D)** R7 inhibits HeLa cell clonogenic formation. **(E)** Clonogenic survival was measured as the number of clones capable of anchorage-dependent growth (right panel). Experiments were performed in triplicate. ^*^P≤0.05, ^**^P≤0.01, ^***^P≤0.001 vs. control.

We investigated the effects of various *P.notoginseng* components on proliferation in various human cancer cell lines (HeLa, MCF7, MCF7/ADR, SW620 and SMMC-7221) via MTT assay. A preliminary screen showed that among the tested components, R7 had the greatest inhibitory effect on HeLa cell viability and proliferation, the results were listed in Supplementary Table 1. Therefore, we chose HeLa cells for continued study. R7 inhibited HeLa cell proliferation in a dose- and time-dependent manner, with an IC_50_ of 10.27±1.84 μM (Figure 1B–1C). Phase contrast microscopy showed that R7 decreased cell densities and increased dead cell numbers (Figure 1D). Colony formation assays confirmed that R7 suppressed HeLa cell proliferation (Figure 1E).

### R7 induces HeLa cell apoptosis

**Figure 2:**
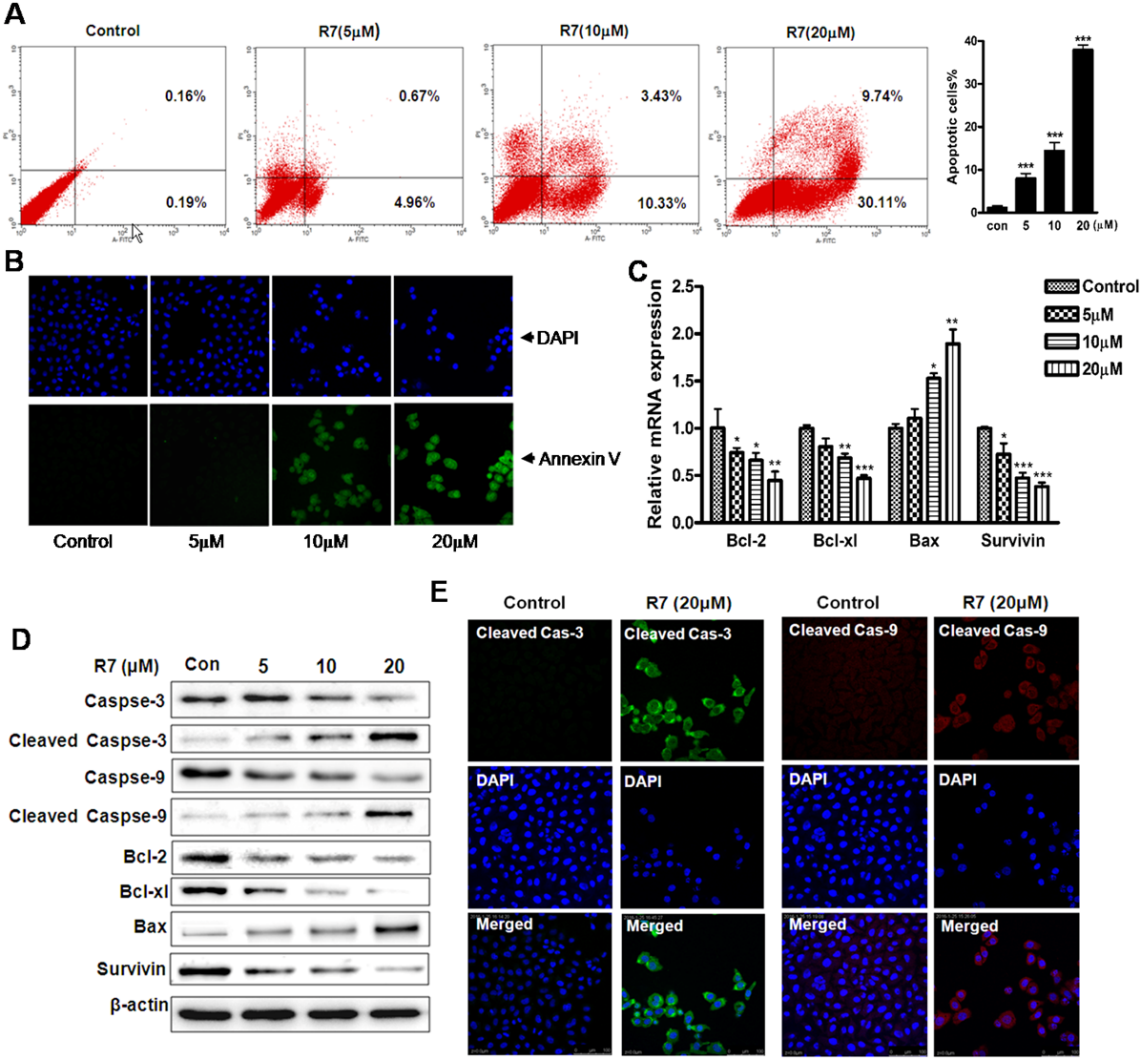
R7 induced HeLa cell apoptosis Cells were treated with various concentrations of R7 for 24 h. Cells were stained with Annexin V-FITC/PI and analyzed via flow cytometry **(A)** and fluorescence microscopy. **(B)** Quantitative Real-Time PCR analysis of Bcl-2, Bcl-xl, Bax, and survivin. **(C)** Western blot analysis of caspase-3/-9, cleaved caspase-3/-9, Bcl-2, Bcl-xl, Bax, and survivin. **(D)** Cleaved caspase-3/-9 was detected using confocal fluorescence microscopy. **(E)** Original magnification: 400×.

HeLa cells treated with R7 were analyzed by flow cytometry to evaluate cell apoptosis. The percentage of HeLa cells undergoing apoptosis increased from 0.35% (untreated) to 39.85% in a dose-dependent manner (Figure 2A). Fluorescence microscopy images confirmed increased apoptosis rates in R7-treated cells (Figure 2B). Quantitative PCR analysis revealed downregulation of anti-apoptotic genes (Bcl-2, Bcl-xl and survivin) and upregulation of the pro-apoptotic gene, Bax, after 24 h R7 treatment (Figure 2C). Immunoblot analysis confirmed these results and showed that R7 induced procaspase-3 and -9 cleavage (Figure 2D, Supplementary Figure 1). Immunofluorescent staining showed increased activated cleaved caspase-3 and -9 forms after R7 treatment (Figure 2E). Together, these results demonstrate that R7 induced apoptosis in HeLa cells.

### Computational modeling of R7-Akt binding

**Figure 3:**
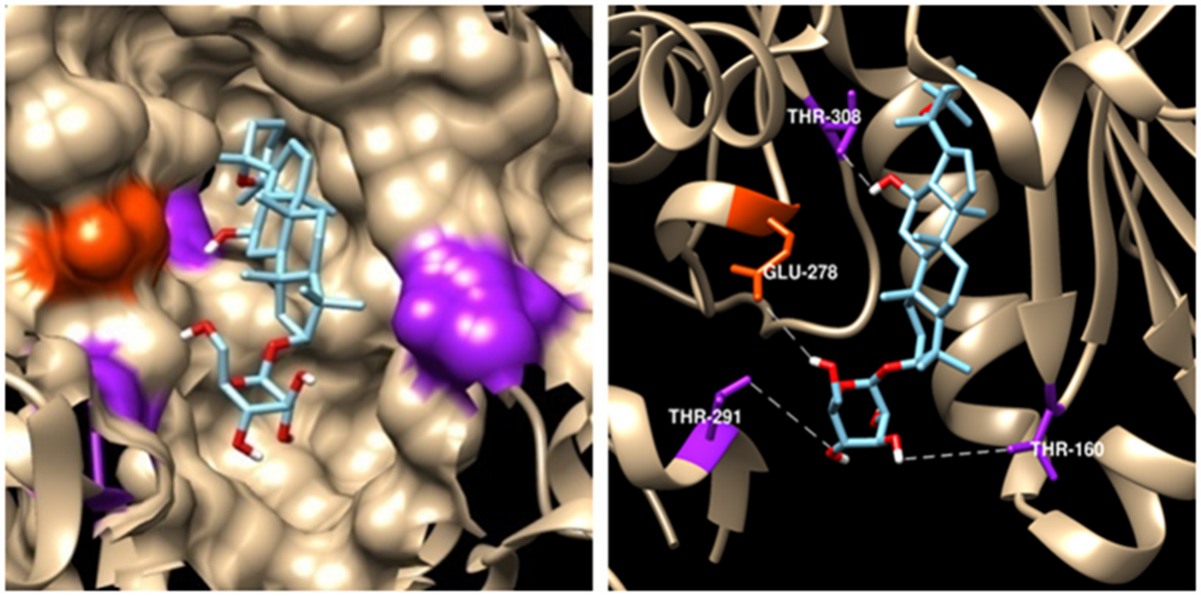
Predicted R7-Aktbinding mode Model of R7 binding to the Akt SMR-binding site (PDB ID code 3DEK). Residues Thr-308, Glu-278, Thr-291, and Thr-160, shown in purple and orange, respectively, on the Akt surface, have the highest pair-interaction energy with R7 (left panel). Four hydrogen bonds were formed with residues Thr-308, Glu-278, Thr-291, and Thr-160. Hydrogen bonds were determined by hydrogen donor-acceptor distance ≤2.9Å and donor-hydrogen-acceptor ≤90° (right panel).

An *in silico* docking-based screening assay was employed to explore mechanisms of R7-induced apoptosis. AutoDock Vina predicted that R7 selectively binds the Akt active binding domain (PDB codes: 3QKL, complexed with the inhibitor, N-{(2S)-3-[(3S)-8',9'-dihydro-1H,3'H-spiro [piperidine-3,7'-pyrano [3,2-e] indazol]-1-yl]-2-hydroxypropyl}-N-(2-ethoxyethyl)-2,6-dimethylbenzenesulfonamide, SMR) among 200 potential targets closely related to pathological tumor processes from the TTD (Therapeutic Target Database) and PDTD (Potential Drug Target Database), the top 10 lowest predicted binding energies (in kcal/mol) of R7 and potential targets from TTD were listed in Supplementary Table 2. The lowest docking energy for R7-Akt (-9.1kcal/mol) was almost equivalent to the binding energy of Akt with SMR (-10.1kcal/mol). Figure 3A shows the best binding model. Surface and cartoon interaction diagrams of the crystal ligand and R7 with Akt revealed that R7 binds Akt active sites and showed strong hydrogen bonding interactions with important amino acids, including Thr160, Thr278, Glu278 and Thr 308 (Figure 3B). As PI3K/Akt signaling plays a central role in carcinogenesis and is frequently targeted by anti-tumor agents [29–32], we speculate that R7 may induce apoptosis by interfering with PI3K/Akt signaling.

### R7 inhibits cancer-related PI3K/PTEN/Akt/mTOR signaling

**Figure 4:**
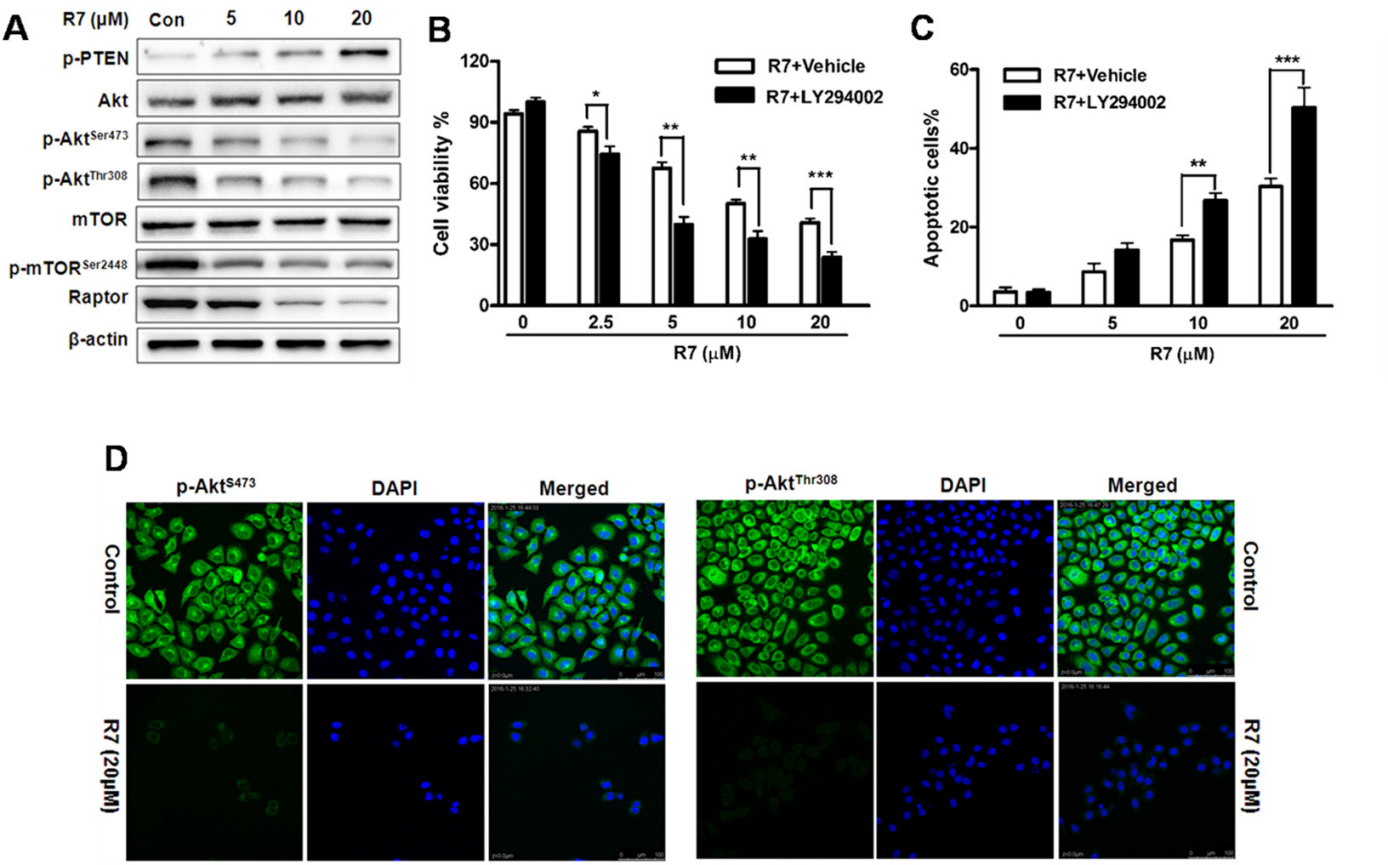
R7 inhibits cancer-related PI3K/PTEN/Akt/mTOR activation in HeLa cells Following R7 treatment, p-PTEN, Akt, p-Akt^Ser473^, p-Akt^Thr308^, mTOR, p-mTOR ^Ser2448^, and raptor were evaluated via western blotting. **(A)** Cells were treated with R7 with or without LY294002 (5 μM) pretreatment, and cell viability was determined by MTT assay. **(B)** Apoptosis induction was analyzed via flow cytometry. **(C)** p-Akt^Ser473^ and p-Akt^Thr308^ levels were assessed using fluorescence microscopy. **(D)** Original magnification: 400×.

We investigated the effects of R7 on PI3K/Akt signaling. Western blotting showed decreased Akt phosphorylation (p-Akt^Ser473^, and p-Akt^Thr308^) and increased p-PTEN (Figure 4A, Supplementary Figure 2) with R7 treatment. p-mTOR^Ser2448^ and raptor were also downregulated dose-dependently (Figure 4A). Immunofluorescent staining and confocal microscopy confirmed p-Akt^Ser473^ and p-Akt^Thr308^downregulation after R7 treatment (Figure 4D). These results indicate that R7 inhibits Akt and mTOR phosphorylation and activates PTEN. MTT and Annexin V-FITC/propidium iodide (PI) assay results showed that pretreatment with the PI3K/Akt inhibitor, LY294002, synergistically enhanced R7 inhibition of HeLa cell proliferation and apoptosis (Figure 4B–4C). These results showed that R7 inhibited aberrant PI3K/PTEN/Akt/mTOR signaling activation.

### *In vivo* R7 anti-tumor efficacy

**Figure 5:**
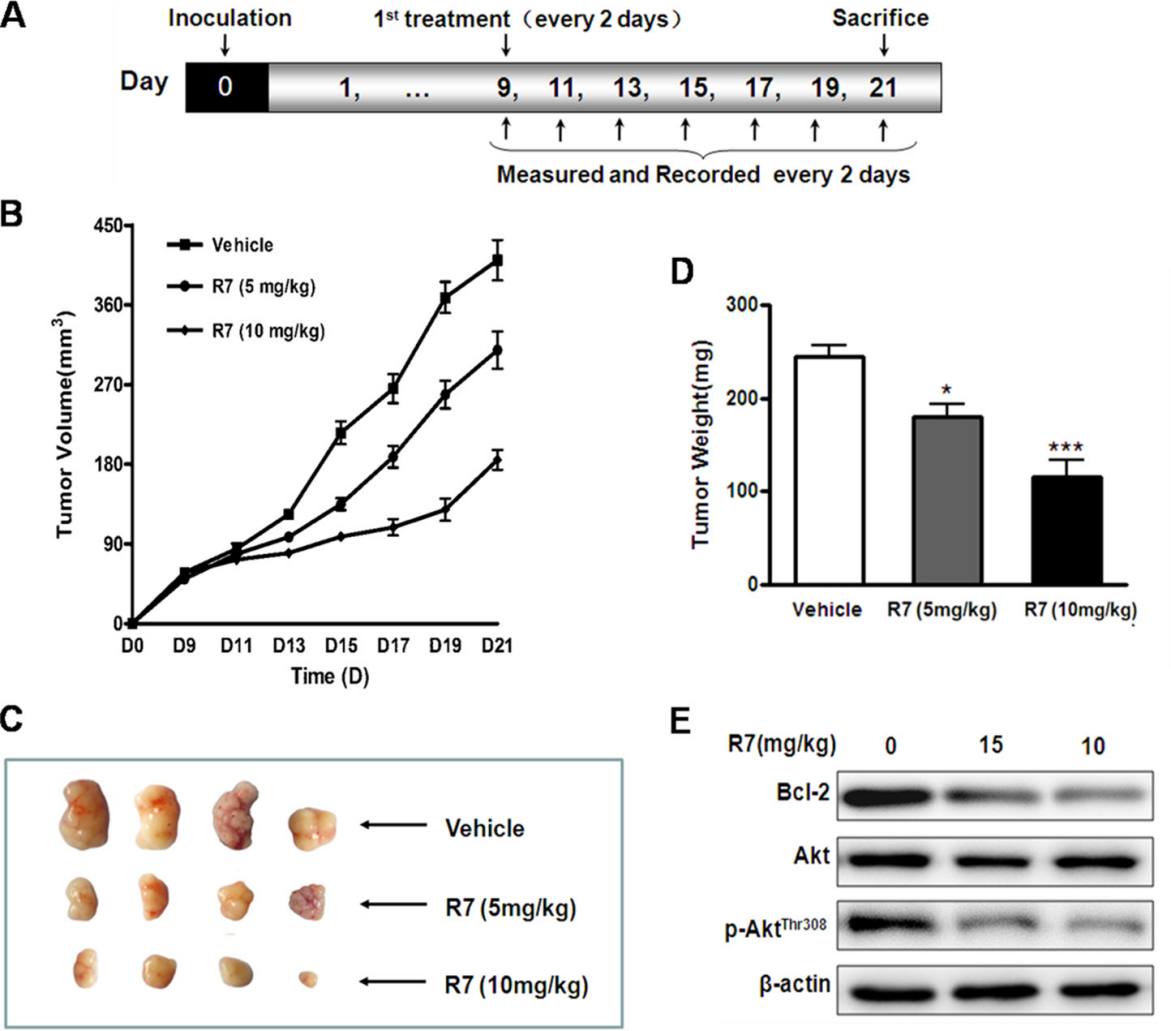
*In vivo* R7 anti-tumor efficacy HeLa cell mouse xenograft model. **(A)** BALB/c male athymic mice were inoculated with 1×10^7^ cells subcutaneously on d 0. R7 (5 or 10 mg/kg/day) or vehicle was administered intraperitoneally once every 2 d from d 9–21, and mice were sacrificed on d 21. Tumor volumes were monitored and tumor volume curves recorded once every 2 d. **(B)** Representative tumors removed from the indicated treatment groups on day 21. **(C)** Average tumor mass at sacrifice after R7 treatment. **(D)** Bcl-2, Akt, and p-Akt^Thr308^levelsin xenograft tissues were evaluated via western blotting **(E)**.

R7 anti-tumor activity was evaluated using HeLa cell xenografts in BALB/c male athymic mice receiving 5 or 10 mg/kg/day R7 for 21d. Tumor size was reduced in R7 treated mice compared to vehicle-treated controls (Figure 5B–5C). At the end of the experiment, tumor weight was reduced by approximately 28% and 52%, respectively, in 5 and 10 mg/kg/day-treated mice compared with controls (Figure 5D). Consistent with *in vitro* results, western blot analyses of tumor tissues revealed Bcl-2 and p-Akt^Thr308^ downregulation in R7-treated mice compared with controls (Figure 5E, Supplementary Figure 3). R7-treated mice maintained normal weights and showed no significant abnormalities throughout the experiments. These results show that R7 has anti-tumor activity in a mouse xenograft models.

## DISCUSSION

Different parts of the *P. notoginseng* plant exert pro-apoptotic and anti- proliferative activities against various cancer cell lines [7, 33]. The present study found that the *P. notoginseng* compound, R7, inhibited HeLa cell proliferation and induced apoptosis in a dose-dependent manner. This was accompanied by anti-apoptotic Bcl-2, Bcl-xl, and survivin downregulation, and pro-apoptotic Bax upregulation, which together triggered caspase-9 and -3 activation. Previous studies showed that certain *P. notoginseng*-derived compounds, including Rh2, Rg5, and Rg3, could inhibit cancer cell proliferation by inducing apoptosis and inhibiting cell cycle progression [34, 35]. Our findings here indicated for the first time that the *P. notoginseng* compound, R7, has anti-tumor activity and is a potential anti-cervical cancer therapeutic.

We used an *in silico* docking-based screening assay to predict the underlying mechanisms of R7-triggered apoptosis. Using the TTD and PDTD, R7 was found to selectively bind Akt. The PI3K/Akt signaling pathway plays important roles in cell growth, apoptosis, differentiation, metabolism, and resistance to chemotherapeutics. Aberrant activation of this pathway is implicated in poor patient outcomes. We speculate that R7 exerts its anti-tumor effects by interfering with PI3K/Akt signaling. Our immunoblotting results indicated that R7 inhibited Akt phosphorylation at Ser473 and Thr308. Akt phosphorylation at Ser473 is associated with resistance to apoptosis via control of pro-apoptotic protein subcellular localization, and may subsequently enhance Akt phosphorylation at Thr 308. Akt phosphorylation at both locations regulates protein synthesis, cell proliferation, and cell shape [36]. Reduced PTEN activity also leads to Akt hyper-activation, and we found that R7 increased PTEN activation in HeLa cells. Our results also showed that R7 treatment downregulated mTOR Ser2448 phosphorylation without affecting mTOR expression, although regulatory-associated protein of mTOR (raptor) expression declined. mTOR can bind raptor to form the mTORC1 complex, which likely regulates the downstream target, p70S6K, to promote apoptosis [37]. Finally, anti-apoptotic (Bcl-2, Bcl-xL and survivin) proteins were downregulatedand the pro-apoptotic cleavage products of caspase-3 and -9 were upregulated in HeLa cells. These results were consistent with those reported by Xue, *et al.* and Zhang, *et al* [38, 39].

We used the Akt inhibitor, LY294002, to further assess the effects of R7 on PI3K/Akt signaling. Pretreatment with LY294002 synergistically enhanced the R7 anti-proliferation and anti-apoptosis effects in HeLa cells, confirming that R7 acts through the PI3K/Akt pathway, which is consistent with previous studies on the mechanism of action of the anticancer agents [40–42]. R7 treatment also reduced tumor growth in a xenografted mouse model, and tumor tissue assays agreed with our *in vitro* findings.

**Figure 6:**
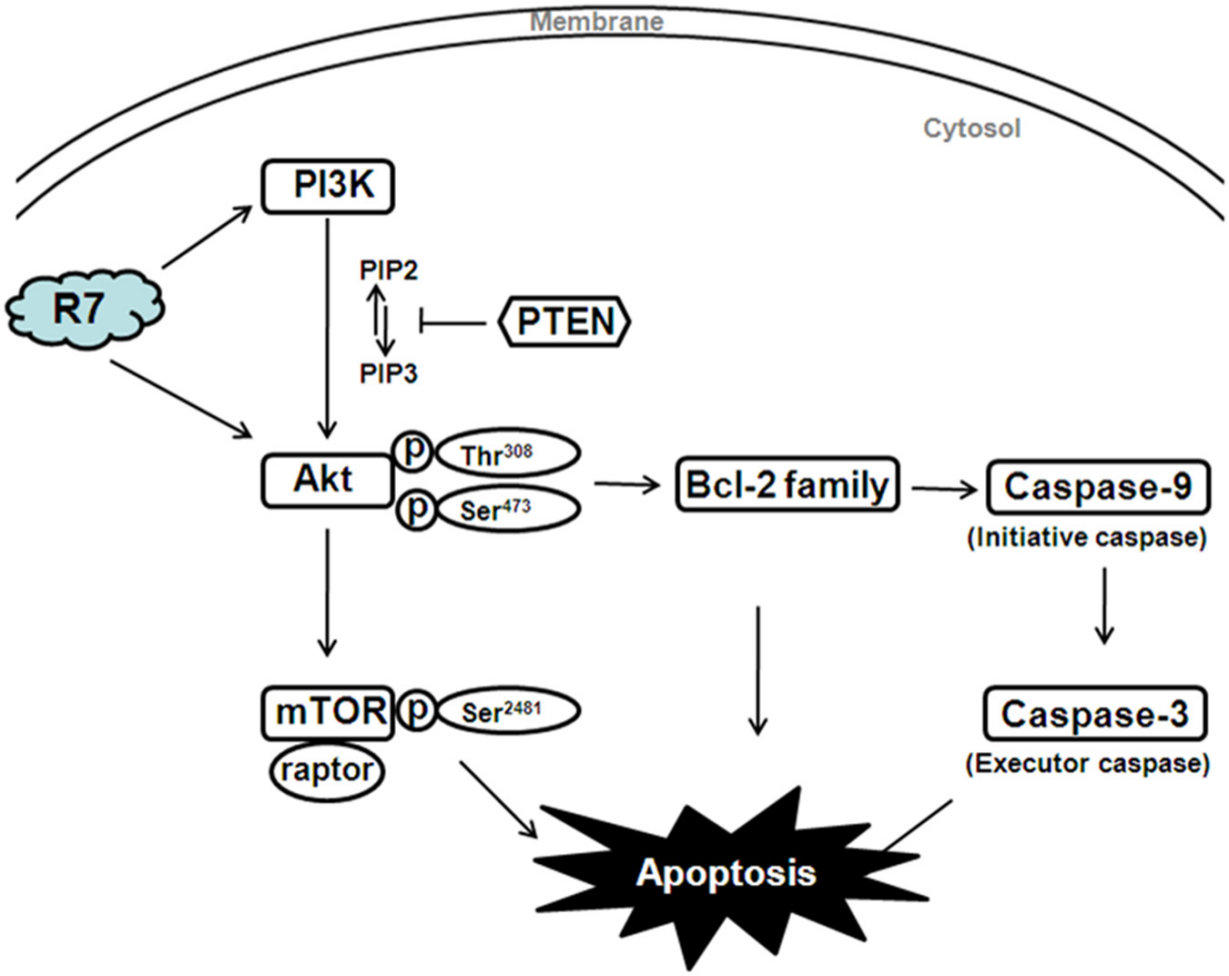
Diagram illustrating the proposed effects of R7 on PI3K/PTEN/Akt/mTOR signaling

In conclusion, our study indicated for the first time that R7 derived from *P. notoginseng* exerts potent anti-tumor activity in HeLa cells by inhibiting cancer- associated PI3K/PTEN/Akt/mTOR activation.R7 targeted both PI3K (PTEN) and Akt, activating the pro-apoptotic Bcl-2 family and, subsequently, caspase family members (Figure 6). While further investigations of the pharmacological effects and molecular mechanisms underlying R7 activity will be required to validate our findings, our results revealed that R7 is a promising chemotherapeutic agent for the treatment of cervical cancer and other PI3K/PTEN/Akt/mTOR signaling-associated tumors.

## MATERIALS AND METHODS

### R7 source

R7 (Figure 1A) is a natural product isolated from the dry root and stem of *Panax notoginseng* (Burk.) [43]. R7 purity was>98% according to HPLC analysis. R7 was dissolved in dimethyl sulfoxide (DMSO) and diluted in cell culture media to a final concentration of ≤0.1%.

### Cell lines and cultures

HeLa cells (human cervical carcinoma cells) were obtained from the American Type Culture Collection (ATCC) and cultured in Dulbecco’s modified Eagle’s medium (DMEM) supplemented with 10% fetal bovine serum (FBS, Gibico, Paisley, UK), penicillin (100 U/ml), and streptomycin (100 mg/ml) at 37°C in a humidified atmosphere with 5% CO_2_.

### Cell viability assay

Cell viability was determined via 3-(4,5-dimethylthiazol-2-yl)-2,5-diphenyl tetrazolium bromide (MTT) assay. Briefly, HeLa cells were seeded and incubated for 24 h, and then either treated for 24 h with 0, 2.5, 5, 10, 20, or 40 μM R7, or treated with R7 at 10 or 20 μM for 0, 12, 24, 48, or 72 h. 100 μL MTT solution (5 mg/mL) was then added to the cells and incubated for 4 h. Absorbance was recorded at 570 nm using an automated spectrophotometric plate reader. Morphological changes were observed under a phase-contrast microscope (Olympus) at 200× magnification and photographed using a digital camera (Nikon, Inc. Japan).

### Clonogenic assay

Cell proliferation was measured using a colony formation assay. Approximately 400 cells/plate were seeded in 6-cm plates, then incubated with R7 at the indicated concentrations for another 24 h. Cells were then washed with PBS and incubated in fresh medium for 15 d. Resulting colonies were fixed in methanol and stained with 1% crystal violet for 0.5 h, and visible colonies were manually counted.

### Cell apoptosis assay

Cell apoptosis was assessed using the Annexin V-FITC/PI apoptosis detection kit (BD Biosciences, San Jose, CA, USA). HeLa cells (5×10^5^/well)were seeded in 6-well plates, treated with R7 or control for 24 h, and then stained using Annexin V-FITC/PI or 1 μg/ml DAPI solution. After incubation for 15 min in the dark, apoptotic cells were analyzed via flow cytometry (Becton Dickinson) or a fluorescence microscope (Olympus). Data were analyzed using CellQuest software (Becton Dickinson).

### *In silico* molecular docking

The 3D structure of R7 was drawn in MOL2 format and converted to Protein Data Bank (PDB) format using ChemOffice 2006 (Chem 3D). Individual PDB files were modified in AutoDock with MGLTools 1.5.6 (Scripps Institute). Crystal structures of the object-protein complexes derived from the TTD and PDTD were obtained from the PDB database. All non-standard molecules (including water molecules, ligands, and other heteroatoms) were removed from the protein structures using Chimera (version 1.10.2). Addition of hydrogen atoms to protein structures was performed using AutoDock (version 1.5.6). For each known ligand type, grid maps were generated that corresponded to each ligand type’s known binding sites on the target proteins.

AutoDock 4.2 and Vina 1.1.2 were used for docking studies. Generally, default settings were used for docking parameters. However, grid spacing was changed from 0.375 to 1.0. The size of the grid was 40×40×40 Å. The internal scoring function was used to assess receptor-ligand interactions. After the standard docking procedure was performed, nine receptor-ligand conformations were generated, and the optimal conformation (least energy) was selected as the active conformation and used to analyze interacting amino acids and hydrogen bonds in ligand-protein complexes.

### Real-time quantitative PCR analysis

HeLa cells were seeded in 12-well dish (1×10^5^/well) and then treated with R7 or control. After 24 h treatment, total RNA was extracted using an RNA extraction kit (Qiagen, Hilden, Germany) and quantified spectrophotometrically. cDNA was prepared using the iScript cDNA Synthesis Kit (Bio-Rad). Real-time PCR amplification reactions were prepared with the SYBR Green PCR Kit (Bio-Rad) and performed using the ABI 7500 Fast Real-Time PCR System (Applied Biosystems). Relative target gene expression was normalized to β-actin and quantified using the 2^-ΔΔCt^ method. Primer sequences were as follows: Bcl-2 forward: 5’-GGATTGTGGCCTTCTTTGAG-3’, reverse: 5’-TACCCAGCCTCCGTTATCCT-3’; Bcl-xl(Bcl-2L1)forward: 5’-CCGATTCATCTACCCTGCTG-3’, reverse: 5’-TCCGC AAAGAACCTGTCAAT-3’; Bax forward: 5’-CCGATTCATCTACCCTGCTGT-3’, reverse: 5’-TGAGCAATTCCAGAGGCAGT-3’; Survivin forward: 5’-GGACCACC GCATCTCTACAT-3’, reverse:5’-CAAGTCTGGCTCGTTCTCAGT-3’; β-actin forward: 5’-CCGCCCTAGGCACCAGGGT-3’, reverse: 5’-GGCTGGGGTGTTGAA GGTCTCAAA- 3’.

### Western blotting

Cells were lysed and equal amounts of cell lysates (15μg) were separated via SDS-PAGE and electroblotted onto PVDF membranes (Bio-Rad). After blocking, membranes were probed with rabbit anti-caspase-3/-9, -cleaved caspase-3/-9, -Bcl-2, -Bcl-xl, -Bax, -phospho(p)-PTEN, -p-Akt^Ser473^, -p-Akt^Thr308^, -Akt, -p-mTOR^Ser2448^, -mTOR, -raptor, and -β-actin antibodies (Cell Signaling Technologies), and blots were visualized using diluted horseradish peroxidase (HRP)-conjugated goat anti-rabbit (mouse) secondary antibodies (Cell Signaling Technologies). After three washes, proteins were detected using the enhanced chemiluminescence (ECL) kit (Millipore, Bedford, MA) and the ChemiGenius Bio-Imaging System (Syngene, Cambridge, UK).

### Immunocytochemistry

HeLa cells treated with R7 for 24 h were fixed with 4% paraformaldehyde (PFA) for 20 min and then permeabilized with 0.2% TritonX-100 in PBS for 20 min. After blocking with 5% bovine serum albumin (BSA) for 60 min, cells were incubated with anti-cleaved caspase-3, -p-Akt^Ser473^, -p-Akt^Thr308^, or -Akt antibody at 4°C overnight and then incubated with Alexa-488-conjugated goat anti-rabbit and Alexa-647- conjugated goat anti-mouse IgG secondary antibodies (1:1500) in darkness for 2 h. Samples were washed and examined under a confocal LSM 510 Laser Scanning microscope (Zeiss, Göttingen, Germany).

### Mouse tumor xenograft model

BALB/c male athymic mice (4–6 weeks old) were maintained under standard pathogen-free conditions, with sterile food and water supplied *ad libitum.* All procedures were conducted in accordance with the Guide for the Care and Use of Laboratory Animals of the US National Institutes of Health and were approved by the Committee on the Ethics of Animal Experiments of Shenzhen University, China.

The mouse xenograft model was performed as described in Figure 5A. HeLa cells (1×10^7^) were inoculated subcutaneously under the armpit at the beginning of the experiment. Tumor volumes were measured and recorded every 2 d from d 7 and calculated using the following formula: tumor volume = a×b^2^/2 (a: major axis, b: minor axis). Once tumor volumes reached 50–75 mm^3^, mice were randomly divided into three groups (n=6/group). R7 (5 or 10 mg/kg/day) or vehicle was administered intraperitoneally every other day until sacrifice on d 21. Tumor xenografts were then immediately removed, weighed, and photographed. Tumor tissues were stored at -80°C for western blotting.

### Statistical analysis

Data were analyzed via one-way of variance (ANOVA) followed by Dunnett’s test, or Kruskai-Wallis ANOVA on Ranks followed by Dunn’s test for multiple comparisons. Data were expressed as means ± S.E.M. The concentration at which 50% inhibition occurred (IC_50_) was calculated using the median-effect equation. SPSS software (version 16.0) was used for statistical tests. P≤0.05 was considered a significant difference.

## SUPPLEMENTARY MATERIALS FIGURES AND TABLES


