# Supplementary material for: Notoginsenoside R7 suppresses cervical cancer via PI3K/PTEN/Akt/mTOR signaling
Source: Oncotarget. 2017 Nov 27;8(65):109487–96. doi: 10.18632/oncotarget.22721 (PMC5752536; doi:10.18632/oncotarget.22721)
Supplement: Supplementary file 1 [file oncotarget-08-109487-s001.pdf]

Notoginsenoside R7 suppresses cervical cancer via PI3K/PTEN/Akt/mTOR signaling

SUPPLEMENTARY MATERIALS

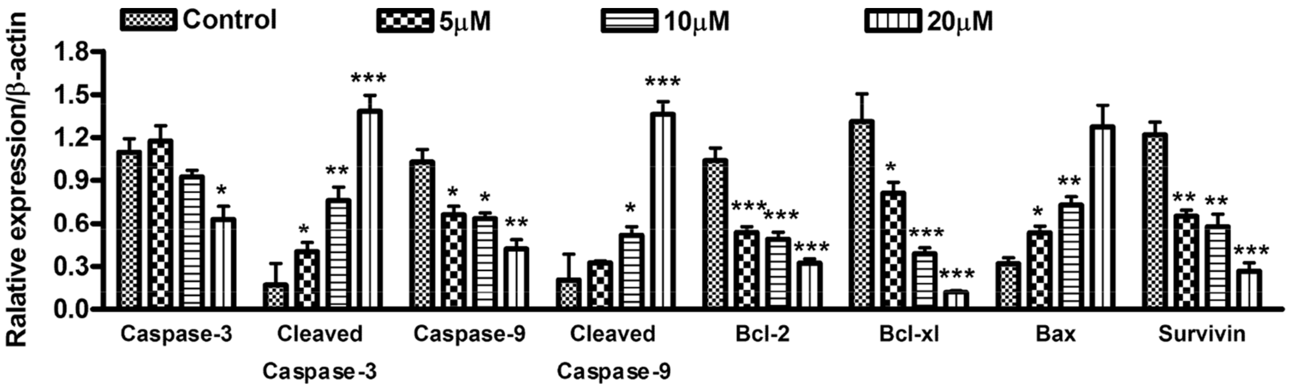

Supplementary Figure 1: Densitometric of western blotting bands in Figure 2D in manuscript were analyzed and expressed relative to β-actin. Data are typical of three experiments and the histogram values are mean ± S.D. *P<0.05, **P<0.01, ***P<0.001, relative to the control group.

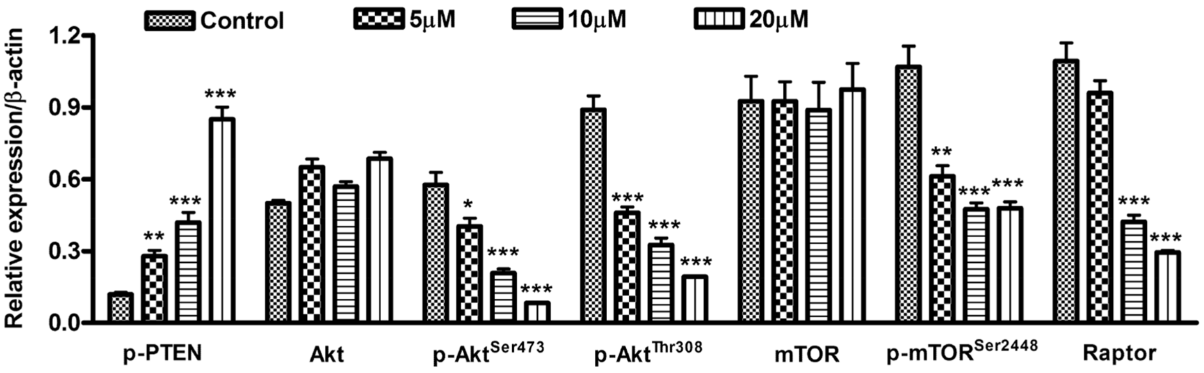

Supplementary Figure 2: Densitometric of western blotting bands in Figure 4A in manuscript were analyzed and expressed relative to β-actin. Data are typical of three experiments and the histogram values are mean ± S.D. $^{*}P<0.05$, $^{**}P<0.01$, $^{***}P<0.001$, relative to the control group.

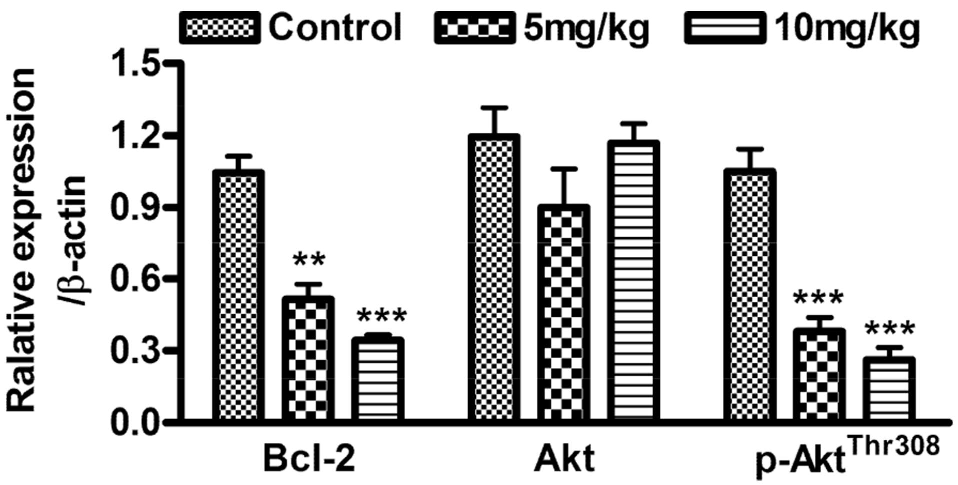

Supplementary Figure 3: Densitometric of western blotting bands in Figure 5E in manuscript were analyzed and expressed relative to β-actin. Data are typical of three experiments and the histogram values are mean ± S.D. $^{***}P<0.001$, relative to the control group.

Supplementary Table 1: The half maximal inhibitory concentration (IC_{50}, μM) of 9 components from *Panax notoginseng* on cell proliferation in 5 human cancer cell lines

	Hela	MCF7	MCF7/ADR	SW620	SMMC-7221
Ginsenoside Rh_1	24.15±1.50	36.48±4.53	369.28±52.91	139.03±17.92	444.55±57.32
Ginsenoside Re	25.43±2.77	77.64±10.02	30.86±5.94	59.96±12.71	121.17±17.36
Ginsenoside Rb	50.54±5.39	42.50±6.04	187.11±9.27	46.90±8.81	169.4±6.31
Ginsenoside Rg_1	128.30±9.48	241.50±34.84	83.68±9.08	170.23±21.95	78.50±9.475
Ginsenoside Rg_2	297.00±28.29	95.34±8.25	233.4±26.08	478.04±29.73	193.33±33.77
Notoginsenoside R_1	20.62±3.97	54.14±6.56	67.17±20.23	59.39±14.89	116.32±18.66
Notoginsenoside R_2	24.48±8.36	42.19±7.65	76.44±9.31	66.78±9.18	84.49±7.48
Notoginsenoside R_7	10.27±1.84	23.91±4.45	25.43±7.94	65.78±10.05	204.07±13.45
Notoginsenoside R_{10}	71.44±9.93	33.73±7.10	51.39±7.55	71.80±20.88	345.51±40.59

[*] Data were obtained from triplicate measurement.

Supplementary Table 2: The top 10 lowest predicted binding energies (in kcal/mol) of R7 and potential targets from TTD

PDB code	Name	Docking energy
3QKL	Akt	-9.1
3H9O	PDK-1	-8.0
3BDY	VEGF	-7.4
3LN1	COX-2	-7.2
3G60	P-gp	-6.9
4QUE	Caspase-3	-6.5
3WF7	S6K1 k	-6.3
3UEG	Survivin	-6.3
3IBF	caspase-7	-5.9
1EJ4	4E-BP1	-5.5
